# 67. Agreement Among Bayesian Dosing Software for Calculating Vancomycin Area Under the Curve

**DOI:** 10.1093/ofid/ofab466.067

**Published:** 2021-12-04

**Authors:** Ross Pineda, Meganne Kanatani, Matthew R Davis, Myung-Shin Sim, Jaime Deville, Christine Pham

**Affiliations:** 1 Ronald Reagan UCLA Medical Center, Los Angeles, California; 2 University of California, Los Angeles, Los Angeles, CA; 3 UCLA Ronald Reagan Medical Center, Los Angeles, California; 4 UCLA Department of Medicine, Los Angeles, California; 5 David Geffen School of Medicine - Department of Pediatrics, Los Angeles, California; 6 University of California, Los Angeles; David School of Medicine/University of California, Los Angeles, Los Angeles, California

## Abstract

This abstract has been withdrawn.

